# Benign Mediastinal Teratoma with Intrapulmonary and Bronchial Rupture Presenting with Recurrent Hemoptysis

**DOI:** 10.5812/iranjradiol.11724

**Published:** 2013-05-20

**Authors:** Farheen Badar, Shagufta Yasmeen, Nishat Afroz, Nazoora Khan, Shah F. Azfar

**Affiliations:** 1 Department of Radiodiagnosis, J.N. Medical College, A.M.U, India; 2 Department of Pathology, J.N. Medical College, A.M.U, India; 3 Firoz Hospital, Aligarh, India

**Keywords:** Mediastinal Teratoma, Rupture, Hemoptysis, Tomography, X-Ray Computed

## Abstract

Mediastinal teratomas are usually asymptomatic tumors, located most commonly in the anterior mediastinum. Very rarely, such tumors may rupture into the tracheobronchial tree, lung, pleura or pericardium. Computed Tomography (CT) is helpful in the diagnosis and differentiation of ruptured and unruptured tumors.

We report a case of ruptured anterior mediastinal teratoma in a 20-year-old female presenting with recurrent hemoptysis and cough; thus, mimicking a lung malignancy or tuberculosis. CT demonstrated a heterogeneous fat containing lesion in the anterior mediastinum with extension into the lingular lobe. Subsequent fine needle aspiration cytology (FNAC) yielded plenty of anucleate squames and debris, and a clear cut diagnosis could not be made. Total excision of the tumor was performed and histopathology of the surgically excised mass confirmed the CT diagnosis.

## 1. Introduction

Teratomas are the most common germ cell tumors of the mediastinum (1). Mostly, they are seen in young adults and are asymptomatic (2). On rare occasions, teratomas may rupture into adjacent structures and give rise to symptoms like dyspnea, chest pain, hemoptysis and fever (1). Herein, we present a case of rupture of the anterior mediastinal teratoma in a 20-year-old female, presenting with recurrent hemoptysis and cough which initially mimicked tuberculosis. Further radiological workup and histopathology confirmed combined intrabronchial and pulmonary extension. To the best of our knowledge, only few such cases are reported in literature. It is important that radiologists are familiar with the CT findings, since it can be the first clue to the proper diagnosis.

## 2. Case Presentation

A 20-year-old female presented with complaints of frank hemoptysis for five days. In addition, for the past three years she had recurrent episodes of hemoptysis and cough, for which she was given repeated courses of antibiotics. Recently she completed the full course of antitubercular treatment from a primary healthcare physician. Previous chest radiographs were reviewed that showed persistent opacity in the left paracardiac region (Figure 1).

**Figure:**
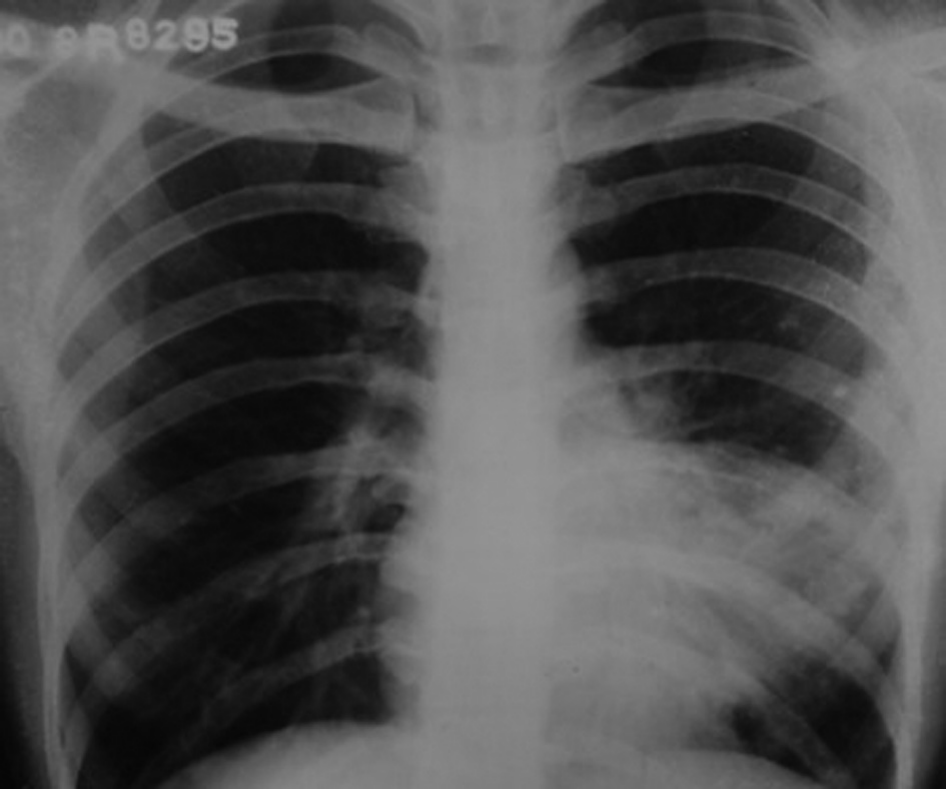
Figure 1. Chest radiograph three years prior to admission shows a large ill-defined opacity with multiple lucencies adjacent to the left heart border

The patient was admitted and a thorough diagnostic workup was done. Routine hematological investigations were unremarkable and the sputum test was negative for acid fast bacilli (AFB). Chest CT revealed a heterogeneous mass with an irregular margin, 3.7 × 4.8 cm in size, involving the lingular lobe and the left mediastinum (Figure 2A). The mass had inhomogeneous density with fat, soft tissue and foci of calcification suggesting the diagnosis of mediastinal teratoma. Areas of consolidation and ground-glass opacities were noted in the lingula of the left lung (Figure 2B).

**Figure:**
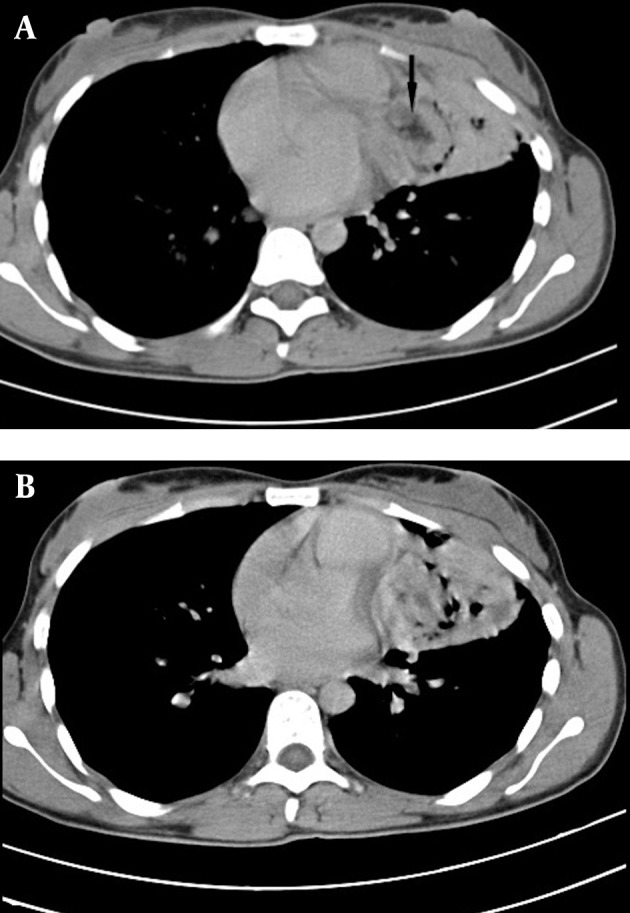
Figure 2. Contrast enhanced axial CT of thorax A, Heterogeneous mass containing fat (arrow), soft tissue, and gas, involving the mediastinum and lingular lobe B, Consolidation and ground-glass opacities seen in lingular lobe adjacent to the tumor

Presence of air within the tumor with narrowing of the lingular lobe bronchus suggested the possibility of communication with the bronchus. CT guided FNAC of the tumor was performed and the cytological findings were inconclusive and there was suspicion of squamous cell carcinoma. Lobectomy of the left lingular lobe with total excision of the tumor was performed. At surgery, a brown tumor was seen in the mediastinum extending to involve the adjacent lingular lobe of lung, with adhesion to the thymus and pericardium. Tumor extension into the bronchial tree was also found. Macroscopy revealed a ruptured cystic structure (4 × 3.5 cm) with a bunch of hairs (Figure 3). Microscopy demonstrated respiratory epithelium, apocrine and eccrine sweat glands, stratified squamous epithelium (Figure 4A) and mesodermal element (Figure 4B) consistent with the diagnosis of a mature cystic teratoma. No immature elements or malignant cells were identiﬁed. Tumor extension into the resected bronchial tree and adjacent lung parenchyma was also found.

**Figure:**
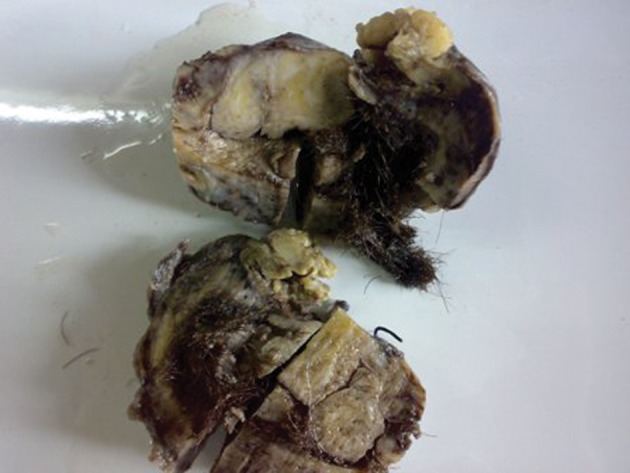
Figure 3. Teratoma gross-cut surface showing predominantly solid and few cystic areas and a bunch of hairs

**Figure:**
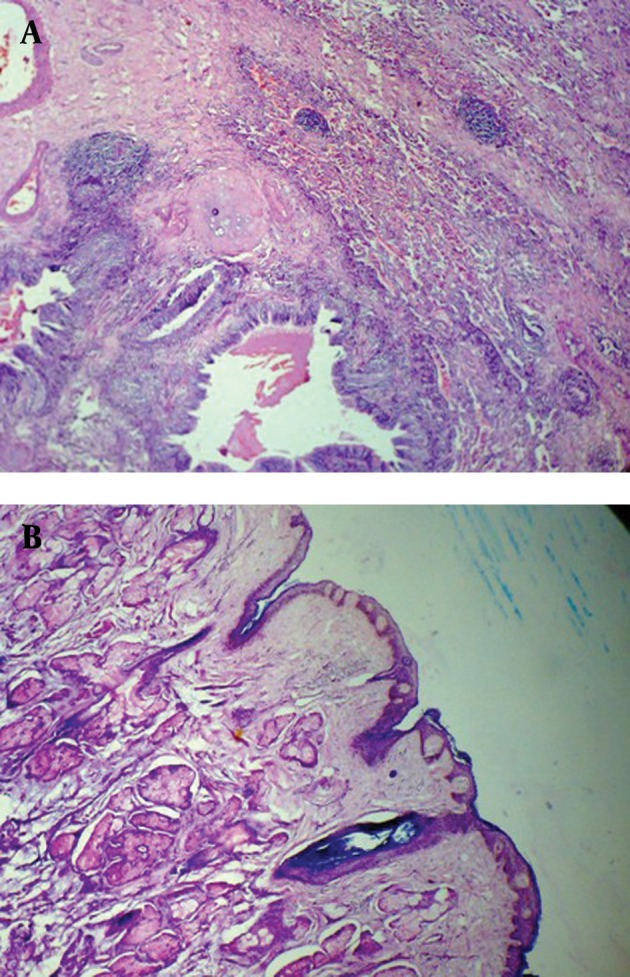
Figure 4. Teratoma tissue section A, Mixture of gland, cartilage, and mesenchymal tissue B, Another area showing ectodermally derived tissue (skin and adnexa) (H & E × 200)

## 3. Discussion

Mediastinum is the most common extragonadal primary site of germ cell tumors, teratoma being the commonest of all mediastinal germ cell tumors. In the mediastinum, teratomas are most frequently located in the anterior compartment (3). They are classified as benign (mature) that consist of well differentiated ectodermal, endodermal and mesenchymal elements, and malignant (immature) that consist of immature tissue as well (4). They are seen in all age groups, but are more common in young adults (3). Benign lesions have no sex predilection; however, malignant ones are more common in males. Mediastinal teratomas rarely produce symptoms except when they attain large size (5) or may rupture into the lung and bronchial tree, pleural space, pericardial space, or great vessels (6, 7). Communication with the tracheobronchial tree gives rise to hemoptysis and trichoptysis while intrapulmonary invasion presents with chest pain, dyspnea, cough and fever (8). All these symptoms may mimic malignancy or pulmonary tuberculosis, especially in TB-endemic countries. In our case also, the presenting complaints of cough and hemoptysis in a young patient was initially misdiagnosed as tuberculosis. On CT, teratoma is seen as smooth or lobulated, heterogeneous solid cystic lesions and the presence of a combination of fluid, soft tissue, calcium and fat is highly specific (3). In our case also, CT revealed a heterogeneous solid mass with fat and calcification. The cystic component was not demonstrated that could be explained because of tumor rupture. CT is of great value not only in the diagnosis of teratoma, but also in the differentiation of ruptured and unruptured tumors (8). This is important for better surgical planning as rupture induces inflammation and adhesions in adjacent structures. Various signs of rupture have been described on CT, which depends on the site, including ill-defined tumor margins, heterogeneous internal components, bursting configuration of internal fat and dirty mediastinal fat (1, 5, 8). There are other signs that are unique to the site of rupture. Rupture into the lung parenchyma can cause chemical pneumonia associated with pleural effusion, sometimes producing an abscess. Imaging findings often resemble those of bacterial pneumonia. Rupture into the pleural or pericardial space may result in effusions and pulmonary edema (9). In our case, heterogeneous appearance of the mass can be explained by rupture, while presence of air foci within the lesion and consolidation in the adjacent lingula imply intrabronchial and parenchymal extension, respectively. The etiology and predisposing factors for rupture still remain controversial and no correlation have been found between tumor size, wall thickness and the probability of rupture (10). Parenchymal consolidation in ruptured teratomas can occur due to obstructive pneumonitis secondary to bronchial invasion; however, intraparenchymal tumor infiltration is also possible (1). Although mature cystic teratoma is histopathologically benign, surgical resection is recommended owing to its complications and potential to rupture. Internal components of the tumor are known to incite inflammation and adhesion; therefore, surgery of the ruptured tumors is often more complicated. Hence, an accurate preoperative radiological diagnosis and pathological confirmation is essential for better surgical planning of the mediastinal teratoma with atypical clinical presentation.
